# Complete genome sequence of a novel classical swine fever virus subgenotype 1.1 detected from a live Japanese encephalitis virus vaccine in South Korea

**DOI:** 10.1128/mra.01120-24

**Published:** 2025-01-14

**Authors:** Guehwan Jang, Changhee Lee

**Affiliations:** 1 College of Veterinary Medicine and Virus Vaccine Research Center, Gyeongsang National University, Jinju, South Korea; Queens College Department of Biology, Queens, New York, USA

**Keywords:** CSFV, contaminated JEV vaccine, complete genome, phylogenetic analysis

## Abstract

A novel classical swine fever virus (CSFV) strain GNU-240601 was identified from a commercial live Japanese encephalitis virus (JEV) vaccine. The whole-genome sequence of GNU-240601 shared the highest similarity with strains belonging to subgenotype 1.1. This is the first identification and complete genome sequence of CSFV from the JEV vaccine.

## ANNOUNCEMENT

Classical swine fever virus (CSFV) is a highly communicable and socioeconomically significant viral pathogen of pigs, notifiable to the World Organisation for Animal Health (1). CSFV (*Pestivirus suis*) is an enveloped, positive-strand RNA virus in the genus *Pestivirus*, family *Flaviviridae* (2). Due to its high genetic variability, CSFV is divided into three major genotypes (1–3) with at least 11 subgenotypes (1.1–1.4, 2.1–2.3, and 3.1–3.4) (1). The CSFV genome is about 12.3 kb, containing a 5′-noncoding region (NCR), a single large open reading frame (ORF), and a 3′-NCR.

In May 2024, a commercial live vaccine against Japanese encephalitis virus (JEV) for sow herds tested positive for CSFV using a VDx CSFV qRT-PCR kit (Median Diagnostics, Chuncheon, South Korea) during pestivirus surveillance on Jeju Island, South Korea. In this study, we report the complete genome sequence of the CSFV strain GNU-240601 detected from CSFV-positive JEV vaccines with the same lot number. Total RNA isolation from the JEV vaccine was performed automatically using an SLA-E13200 TANBead Nucleic Acid Extraction System (Taiwan Advanced Nanotech, Taoyuan, Taiwan). First-strand cDNA synthesis was performed using PrimeScript 1st strand cDNA Synthesis Kit (TaKaRa, Otsu, Japan) and the CSFV specific primers. Seven overlapping fragments covering the entire viral genome were PCR amplified using TaKaRa Ex Taq DNA polymerase (TaKaRa) with previously reported primer sets (3–5). Each PCR amplicon was gel-purified and cloned into pGEM-T Easy Vector (Promega, Madison, WI). The 5′ and 3′ ends of the viral genome were determined via rapid amplification of cDNA ends as described previously (6). Five clones were then sent to a commercial service (SolGent, Daejeon, South Korea) for Sanger sequencing on an ABI 3730XL DNA Analyzer. Nucleotide sequences were assembled and aligned using Geneious Prime 2024.0.7 (Biomatters, Auckland, New Zealand), and the phylogenetic tree was constructed in MEGA X software using the neighbor-joining method (7).

**Fig 1:**
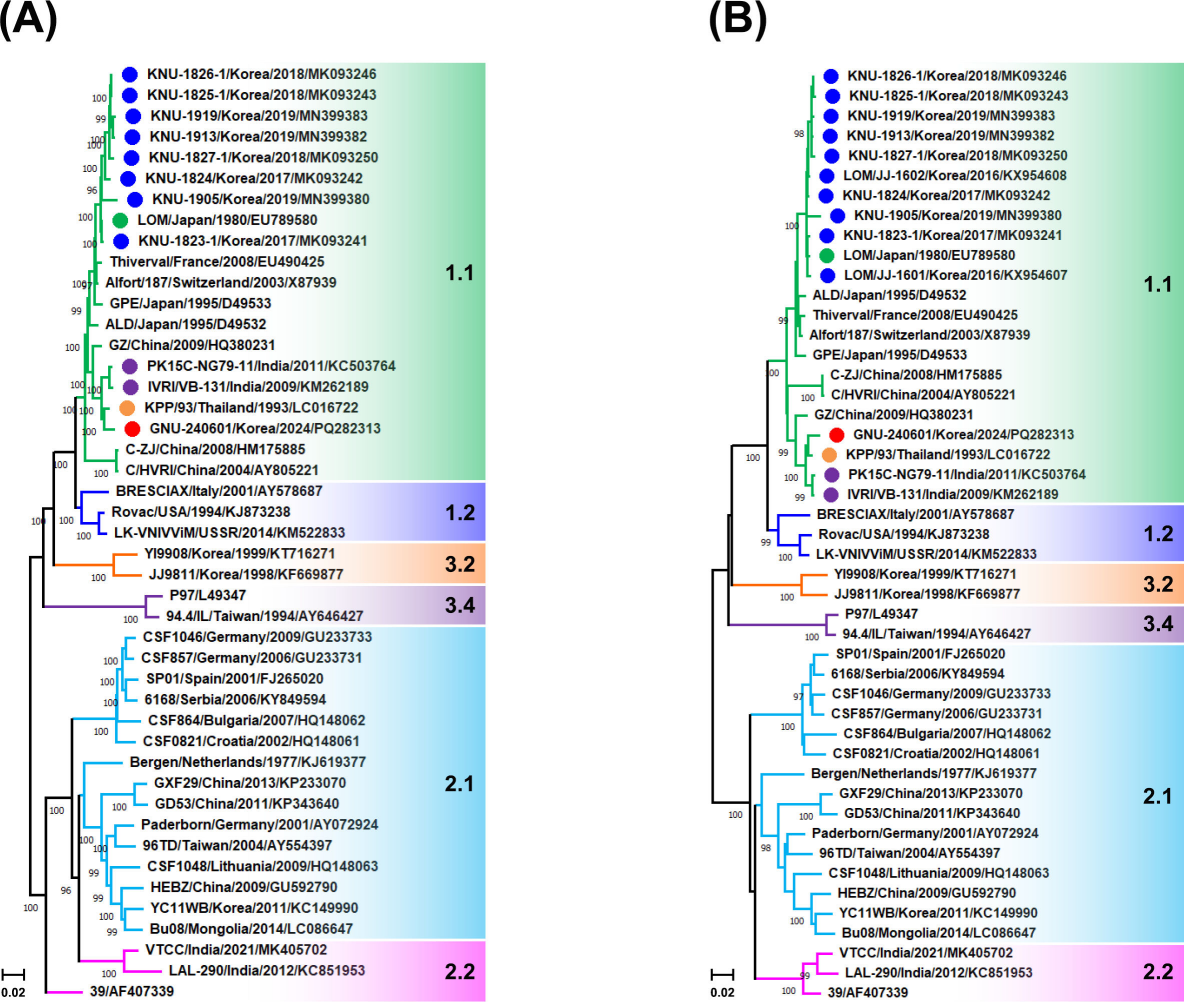
Phylogenetic analysis based on whole-genome (**A**) and E2 gene (**B**) sequences of CSFV strains from NCBI nucleotide database. Multiple sequence alignments were performed using ClustalX, and phylogenetic trees were constructed using the neighbor-joining method in MEGA X software. Numbers at each branch represent bootstrap values greater than 50% based on 1,000 replicates. Strain names, isolation countries and dates (year), GenBank accession numbers, and genotypes are shown. Genotypes are shaded in different colors: green (1.1), blue (1.2), orange-red (3.2), purple (3.4), sky blue (2.1), and pink (2.2). A red circle indicates GNU-240601 identified in this study, a green circle indicates the LOM strain;, blue circles indicate LOM-derived field strains, and orange and purple circles indicate strains from Thailand and India, respectively, showing high homology with GNU-240601. Scale bars indicate nucleotide substitutions per site.

The full-genome sequence of GNU-240601 consisted of 12,297 nucleotides (nt) long (GC content, 46.5%), including a 373 nt 5′-NCR, an 11,697 nt ORF encoding a 3,898 amino acid polyprotein, and a 227 nt 3′-NCR. This was 1-nt shorter than a live attenuated low-virulence Miyagi (LOM) vaccine strain (GenBank accession number EU789580) due to unique 1-nt insertion and 2-nt deletion in 3′-NCR. GNU-240601 shared 96.3% homology with the LOM strain, attributed to mutations of 11 nt in the 5′-NCR, 431 nt in the polyprotein (280 synonymous and 151 non-synonymous), and 14 nt in the 3′-NCR, and 95.1% identity with the LOM-derived field strain KNU-1905 (MN399380) (Table 1). Subsequent nucleotide BLAST analysis found that GNU-240601 exhibited maximum homology of 98.7% with the Thailand KPP/93 strain (LC016722) (8) and 98.0% and 97.9% with two Indian isolates, IVRI/VB-131 and PK15C-NG79-11 (KM262189 and KC503764), respectively (9, 10). Phylogenetic trees depicted in Fig. 1 clustered GNU-240601 within the subgenotype 1.1 clade, grouping with KPP/93, IVRI/VB-131, and PK15C-NG79-11, while being distinct from LOM and its derived field isolates. The detection and whole-genome sequence of GNU-240601 will help identify the potential origin of the vaccine contamination and improve vaccine safety.

**TABLE 1:** Detailed comparison of the whole-genome sequences of GNU-240601 and other genotype-representative strains

Subgenotype	Strain	Nucleotide/amino acid identity (%) (no. of nucleotide/amino acid differences)			
		5′-NCR	Polyprotein	3′-NCR	Total
1.1	LOM/Japan/1980	97.1 (11)	96.3/96.6 (431/131)	94.2 (14)	96.3/96.6 (456/131)
	KNU-1905/Korea/2019	96.5 (13)	95.1/96.0 (570/154)	93.4 (16)	95.1/96.0 (599/154)
	GPE/Japan/1995	97.6 (9)	96.4/96.8 (422/124)	95.5 (11)	96.4/96.8 (442/124)
	GZ/China/2009	97.9 (8)	97.4/97.4 (304/102)	95.0 (12)	97.4/97.4 (324/102)
	KPP/93/Thailand/1993	100 (0)*^a^*	98.7/98.2 (152/67)	NA*^b^*	98.7/98.2 (152/67)
	IVRI/VB-131/India/2009	98.7 (5)	98.0/97.8 (238/85)	97.1 (7)	98.0/97.8 (250/85)
	PK15C-NG79-11/India/2011	98.1 (7)	98.0/97.7 (238/90)	95.9 (10)	97.9/97.7 (255/90)
1.2	Rovac/USA/1994	97.8 (8)	94.3/95.5 (672/176)	82.6 (42)	94.2/95.5 (722/176)
2.1	96TD/Taiwan/2004	91.2 (33)	85.0/91.9 (1,759/315)	73.8 (62)	84.9/91.9 (1,854/315)
2.2	LAL-290/India/2012	92.5 (28)	83.1/88.7 (1,982/441)	74.8 (61)	83.1/88.7 (2,071/441)
3.2	JJ9811/Korea/1999	90.6 (35)	87.8/92.8 (1,431/282)	88.8 (27)	87.7/92.8 (1,493/282)
3.4	94.4/IL/Taiwan/1994	91.7 (31)	85.1/91.9 (1,748/318)	75.6 (59)	85.0/91.9 (1,838/318)

^*a*^ Partial sequences of the KPP/93 strain.

^*b*^ NA, Not available.

## Data Availability

The complete genome sequence of CSFV isolate GNU-240601 was deposited in GenBank under accession no. PQ282313. Raw sequence data were deposited in the NCBI Sequence Read Archive (SRA) under SRA accession no. SRX26687346 and BioProject accession no. PRJNA1184947.
